# Dental Mesenchymal Stem Cell-Based Translational Regenerative Dentistry: From Artificial to Biological Replacement

**DOI:** 10.3389/fbioe.2018.00049

**Published:** 2018-05-02

**Authors:** Mona K. Marei, Rania M. El Backly

**Affiliations:** ^1^Department of Removable Prosthodontics, Faculty of Dentistry, Alexandria University, Alexandria, Egypt; ^2^Tissue Engineering Laboratories, Faculty of Dentistry, Alexandria University, Alexandria, Egypt; ^3^Endodontics, Conservative Dentistry Department, Faculty of Dentistry, Alexandria University, Alexandria, Egypt

**Keywords:** prosthodontics, biological replacement, dental pulp stem cells, peri-implantitis, translational regenerative dentistry

## Abstract

Dentistry is a continuously changing field that has witnessed much advancement in the past century. Prosthodontics is that branch of dentistry that deals with replacing missing teeth using either fixed or removable appliances in an attempt to simulate natural tooth function. Although such “replacement therapies” appear to be easy and economic they fall short of ever coming close to their natural counterparts. Complications that arise often lead to failures and frequent repairs of such devices which seldom allow true physiological function of dental and oral-maxillofacial tissues. Such factors can critically affect the quality of life of an individual. The market for dental implants is continuously growing with huge economic revenues. Unfortunately, such treatments are again associated with frequent problems such as peri-implantitis resulting in an eventual loss or replacement of implants. This is particularly influential for patients having co-morbid diseases such as diabetes or osteoporosis and in association with smoking and other conditions that undoubtedly affect the final treatment outcome. The advent of tissue engineering and regenerative medicine therapies along with the enormous strides taken in their associated interdisciplinary fields such as stem cell therapy, biomaterial development, and others may open arenas to enhancing tissue regeneration via designing and construction of patient-specific biological and/or biomimetic substitutes. This review will overview current strategies in regenerative dentistry while overviewing key roles of dental mesenchymal stem cells particularly those of the dental pulp, until paving the way to precision/translational regenerative medicine therapies for future clinical use.

## Introduction

Prosthodontics is “the dental specialty pertaining to the diagnosis, treatment planning, rehabilitation and maintenance of the oral function, comfort, appearance, and health of patients with clinical conditions associated with missing or deficient teeth and/or maxillofacial tissues using biocompatible substitutes” (Academy of Prosthodontics, 2005). Through the years, materials and techniques have improved to enhance the outcome of appearance, mastication, phonetics, and function of millions of patients all around the world (Ellis et al., 2010; Behr et al., 2012; Lynch, 2012). For example, implant-supported dentures have definitely upgraded the level of patient satisfaction over the past 20 years as compared to conventional removable dentures (Sanchez-Siles et al., 2018). The advancement of digital techniques employing computer aided designing and computer aided manufacturing (CAD/CAM) has also enhanced the ease and precision of replacement prosthetics such as removable partial dentures (Ye et al., 2017). Computer-aided designing techniques have also paved the way to major improvements in the surgical planning and placement of implants and have increased the precision and as such the predictability of prosthetic treatment outcomes (Schnutenhaus et al., 2018).

Although such conventional artificial replacements are continuously being challenged there is still much room for advancements of such therapies as they will continue to serve thousands if not millions of people particularly of the aging population in the upcoming years. The introduction of zirconia implants has also had a pronounced effect on enhancing the quality of implant-retained prosthetics. A recently published retrospective clinical study has shown that the rehabilitation of edentulous jaws using complete-arch fixed implant-supported zirconia prosthesis with veneered porcelain displayed high survival rates for both the prostheses and the implants with minor complications encountered (Tischler et al., 2018). Current advancements in artificial replacement strategies have also extended to other facial prostheses for management of auricular defects which comprise a major sector of maxillofacial deformities. Again the use of CAD/CAM techniques has greatly facilitated obtaining an auricular prosthetic replacement therapy that is accurate, requires fewer number of visits for the patient and which can have a great impact on improving patients' quality of life (Yadav et al., 2017).

However, although dental replacement therapy is considered an economic and easy treatment, however, this kind of therapy is associated with various complications resulting in failure, frequent repair, and does not always allow physiological function of dental and oral-maxillofacial tissues, all of which are critical factors that affect quality of life (Bilhan et al., 2012; Dewan et al., 2015). Current therapeutic interventions in dentistry are based on sophisticated biomaterials and dental implants yet, there is an enormous unmet need for all these innovative methods to enable a balance between formation of new dental tissue while maintaining normal physiological function (Albrektsson et al., 2014; Carcuac and Berglundh, 2014; Lindhe and Pacey, 2014; Derks et al., 2016). The Federation of Periodontology has provided a forecast that 80 percent of patients and 50 percent of dental implants will develop peri-implant mucositis in the future. These corresponding figures for peri-implantitis are 28–56 percent of the patients and 12–43 percent of the implants (Mombelli et al., 2012). A meta-analysis of 504 studies which included 1,497 patients with 6,293 implants reported the prevalence of peri-implant mucositis to be 63.4% of patients and 30.7% of implants. Smokers recorded higher frequency of peri-implant diseases of up to 36.3% (Atieh et al., 2013). The promise of patient-specific substitutes to enhance tissue regeneration has provided a new approach toward addressing the issue of periimplantitis in spite the fact that numerous efforts have been made to develop more predictable methods for treatment of this disease (Khoshkam et al., 2013; Larsson et al., 2016).

When the field of cell therapy “known later on as tissue engineering and regenerative medicine” was introduced 30 years ago, no one believed that it would extend to the dental and maxillofacial region, while the more critical organs and tissues were on the top of the priority list e.g., liver, kidney, heart, etc. If we look today, the most successful applications of tissue engineering and regenerative medicine are skin, cornea, bone, dental pulp, periodontal ligament, and alveolar bone; all related to the dental and head and neck area (Jalali et al., 2014; Chen F. M. et al., 2016; Miller et al., 2016).

## The nature of dental mesenchymal stem cells: special focus on the dental pulp reservoir

Most of the tissues in the craniofacial region including the dental pulp and periodontal ligament, are of mesenchymal origin yet specifically derived from the neural crest during embryonic development and so they are frequently termed as ecto-mesenchyme stem cells “EMSC.” These dental EMSC appear not only to be present in permanent teeth but in the deciduous dentition as well particularly in the pulp and periodontium and exist in substantial amounts (La Noce et al., 2014a). In the dental pulp these cells serve to maintain homeostasis by acting as a reservoir for dental pulp fibroblasts, to replace damaged odontoblasts when needed and to produce reparative dentin (Morikawa et al., 2016).

Several similarities exist between dental pulp stem cells (DPSC) and bone marrow-derived mesenchymal stem cells (BMMSC) with both cell types exhibiting a fibroblast-like phenotype in culture and staining positively for mesenchymal cell markers yet negatively for hematopoietic cell markers. Both BMMSC and DPSC have clonogenic and multi-differentiation potentials being able to differentiate into osteogenic, adipogenic, and chondrogenic lineages (Gronthos et al., 2000; Perry et al., 2008). Mesenchymal stem cells have been found to have dual origin; pericytic and non-pericytic. This fact may imply that tissues harboring higher vascularity may provide more stem cells with a “pericytic” origin to the repair process (Feng et al., 2011).

Donor-matching experiments have demonstrated that DPSC contain a bigger population of stem/progenitor cells and have higher population doubling times than BMMSC (Alge et al., 2010). DPSC also had significantly higher alkaline phosphatase activity than BMMSC following osteogenic differentiation, supporting the possibility of using DPSC to regenerate mineralized tissue. Sub-cloned DPSC were shown to retain their ability to proliferate whereas BMMSC did not. As such, researchers have strongly suggested that DPSC populations may possess enhanced features allowing them to be better candidates than BMMSC for tissue engineering applications (Yu et al., 2007; Huang G. T. et al., 2009).

DPSC have also exhibited the ability to differentiate into melanocytes, myocytes, cardiomyocytes, hepatocyte-like cells, and active neurons *in vitro*. Consistently, the reconstruction of large-scale cranial bone defects was reported in non-immunocompromised rats (de Mendonca Costa et al., 2008; Graziano et al., 2008). Additionally, a dental pulp cell/collagen sponge biocomplex was able to completely regenerate mandibular bone defects in patients indicating clear evidence that autologous DPSC could be used as an effective therapeutic strategy for the repair of bone defects with minimal risks involved (d'Aquino et al., 2009). In a follow-up for the clinical cases regenerated by DPSC, it was revealed that the regenerated bone was extremely hard and was actually highly vascularized compact rather than spongy bone which represented a non-physiological nature of the regenerated tissue in relation to that site (Giuliani et al., 2013).

The role of DPSCs in bone regeneration around dental implants has also been investigated and showed that this cell population exhibited the highest osteogenic potential as a source for tissue-engineered bone around titanium implants (Yamada et al., 2010). In more critical cases, it was stressed on the safety of using a graft consisting of DPSC, platelet rich plasma gel and tricalcium phosphate as a scaffold in cases of osteoradionecrosis of the mandible (Manimaran et al., 2014).

Although large numbers of basic scientific and clinical researches have demonstrated the potential of DPSC isolated from 3rd molars or incisors to generate mineralized tissue, dentin, periodontal ligament, or dental pulp; their potential has also extended to applications in orthopedic, oral, and maxillofacial reconstruction (Sonoyama et al., 2006; Aurrekoetxea et al., 2015; Khojasteh et al., 2015; Gao et al., 2016; Li et al., 2016). Recently, the possibility that such cells may have applications beyond the scope of the head and neck region has paved the road toward translational regenerative medicine.

## Dental mesenchymal stem cells beyond regenerative dentistry

### Neuro-regenerative potential of dental mesenchymal stem cells

All types of dental mesenchymal stem cells (MSC) express nestin (neural stem cell marker), in addition to other neural crest stem cell (SC) markers (musashi-1, p75, snail-1,-2, slug, Sox-9, etc.) indicating their embryonic origin. Undifferentiated dental MSCs express markers of both neural stem/progenitor cells and mature neural cells, including SOX-2, tenascin C, ENO-2, MAP2ab, c-FOS, Nestin, Neurofilament (NEF-H and NEF-L), Glial Fibrillary Acidic Protein (GFAP), bIII-tubulin, and Microtubule-Associated Protein 2 (MAP-2) (Osathanon et al., 2014; Pall et al., 2017). Indeed, a significant subpopulation of DPSCs was recently demonstrated to have glial origins which confirms the neuroregenerative potential of these cells (Kaukua et al., 2014).

Recent studies have demonstrated that tooth-driven stem cells promoted strong neuroregenerative activities that fulfill many requirements for functional recovery after spinal cord injury. DPSCs and stem cells from human exfoliated deciduous teeth (SHED) can differentiate into functionally active neurons with the ability to express voltage-gated Na+ channels *in vitro* and *in vivo* toward neuron-like cells within only 48 h of transplantation (Arthur et al., 2008; Martens et al., 2014). DPSC-differentiated Schwann cells have also recently been shown to effectively participate in neural tissue regeneration providing a promising tool for peripheral nerve tissue repair (Sanen et al., 2017).

Multiple mechanisms of action involved in the neuroregenerative potential of these cells have been observed. The first is that these cells could inhibit apoptosis of neurons, astrocytes, and oligodendrocytes, which directly improved the preservation of neuronal filaments and myelin sheaths. Second, they inhibited the expression of multiple axon growth inhibitors such as chondroitin sulfate proteoglycan and myelin-associated glycoprotein, via paracrine mechanisms which directly promoted the regeneration of transected axons. They could then replace the lost cells by differentiating into mature oligodendrocytes (Sakai et al., 2012; Yamagata et al., 2013).

#### Dental mesenchymal stem cells: a fountain of youth

Although mesenchymal stem cells are promising tools for cell-based tissue engineering strategies, the decline in their cellular proliferation, differentiation potential as well as their regenerative ability with increasing donor age is a valid limitation. The vital role of bone marrow MSCs in cell-based therapies is shown through their immunomodulatory, trophic, and paracrine functions that may have the greatest *in vivo* therapeutic impact however, these functions have been demonstrated to be age-dependent (Fafian-Labora et al., 2015).

Though DPSC and BMMSC share many common features, there are differences. The ability to form dental tissues and differentiate into odontoblasts are unique to DPSCs. Investigation into the effects of age on cell source is becoming some important issue especially as older patients become the recipients of procedures for regenerative therapy. With increasing age, the properties of MSCs are altered leading to problems when using autologous MSCs from aged donors for cell-based therapies. Cellular functions of aged BM-MSCs change leading to a reduction in responsiveness to biological and mechanical signals which are related to increased oxidative stress exposure as well as a less dynamic actin cytoskeleton which favor macromolecular damage and senescence. Age-related changes in human MSCs include increases in apoptosis in addition to upregulation of the *p53* pathway as well as decreased proliferation and osteogenic differentiation abilities (Zhou et al., 2008; Kasper et al., 2009).

When compared to BMSCs, research data suggested there is no significant change in the DPSC percentage with age, yet, with aging the amount of present DPSCs in the tooth likely decreases. This is a result of age-related changes leading to reduced volume of pulpal tissue, deposition of dentin internally, dystrophic calcification within the vascular components, and an increase in the fibrous component of the dental pulp. Some studies have shown that with increased age, there is a decrease in the proliferative capacity of DPSCs as well as their osteogenic/dentinogenic potential. Human DPSCs from aged donors appear to lose their proliferative and differentiation capabilities with advanced passaging. Growing human DPSCs under hypoxic conditions under 3% O_2_, appears to have succeeded in reversing this deficiency, indicating the possibility to obtain sufficient amounts of DPSCs from older patients (Gronthos et al., 2002; Iida et al., 2010).

Indeed, although there is a decrease in the proliferative capacity of DPSC by age this can be modulated by the extrinsic microenvironment. Another important matter is that aging can negatively impact neurogenic differentiation in human DSCs, but the activation of Wnt/β-catenin can this reverse the age-associated decline in neurogenic differentiation. This may support the therapeutic application of these cells for treating nerve injury and neurodegenerative diseases (Feng et al., 2013). In a nerve guide tube model of PLGA, DPSCs were able to promoted 7-mm-long facial nerve gap repair *in vivo*. Cultured dental pulp-derived cells produced neurotrophic factors, such as nerve growth factor, brain-derived neurotrophic factor, and glial cell line-derived neurotrophic factor, all of which are key elements to protect from facial motor neuron death and promote peripheral nerve regeneration (Sasaki et al., 2011; Yamamoto et al., 2016).

In a recent study aiming to more precisely identify the MSC populations present in the human dental pulp upon passaging, it was found that there was progressive increase in the number of (CD56-Neural Cell Adhesion Molecule) with passages *in vitro*. CD56 is a cell adhesion molecule which belongs to the superfamily of immunoglobulin receptors. It is abundantly expressed in the central nervous systems and hence it mediates several neuronal functions by controlling intercellular adhesion, neurite outgrowth, and cell migration, proliferation, survival and differentiation; all of which are characteristics of cells migrating from the cephalic neural crest (Ducret et al., 2016).

Mesenchymal stem cells from dental origins other than pulp tissue have been shown to be affected by donor age in regards to proliferation, migration, and differentiation potential as with periodontal stem cells (Zhang et al., 2012). The effect of aging on the regenerative potential of DPSCs has also been investigated thoroughly in an ischemic hindlimb and an ectopic tooth root model in order to determine its influence on the mobilization of specific subpopulations of DPSC when subjected to a granulocyte-colony stimulating factor (G-CSF) protocol. The results indicated that mobilized DPSCs from aged donors were similar to those from young donors in their capacities for migration, differentiation, expression of angiogenic/neurotrophic factors. Their trophic effects on proliferation and migration, and anti-apoptotic effects were also comparable indicating that this population maintains its properties irrespective of age. Pulp mesenchymal stem cells from aged and young donors expressed stem cell markers as CXCR4, GCSFR, and CD105. The mobilization isolation protocol using granulocyte-colony stimulating factor allowed DPSCs to overcome the decrease in stemness occurring with age (Horibe et al., 2014).

#### Dental stem cell-paracrine-mediated functions for regenerative applications

These previously mentioned characteristics suggest an immense utility for stem cells from the dental pulp in clinical applications in regenerative dentistry as well as ischemic diseases by autologous cell transplantation. DPSCs were able to induce cardiac repair in the absence of cell differentiation, which occurred through an increase in vessel numbers and a reduction in the size of the infarcted area as it was demonstrated before with bone marrow mesenchymal stem cells. Their ability to secrete proangiogenic VEGF improved cardiac function by reversing the cardiac ratio of angiopoietin-1 to angiopoietin-2 and antiapoptotic factors (Gandia et al., 2008). Human DPSC-induced paracrine-mediated angiogenesis took place via production of high amounts of angiogenic molecules, stimulation of endothelial cell migration by activation of the P13l–AKT and MEK–ERK pathways and thus significantly induced the formation of blood vessels, highlighting the suitability of DPSCs for treatment of stroke and myocardial infarction; diseases in which a reduction of angiogenesis is clear (Tatullo et al., 2015).

Paracrine-mediated angiogenesis by DPSCs is associated with the secretion of a broad range of regulatory proteins such as platelet derived growth factor (PDGF), basic fibroblast growth factor (bFGF), and vascular endothelial growth factor (VEGF), either in normal conditions or in response to noxious stimuli, such as injury or hypoxia. Other angiogenesis-promoting factors that have been detected in DPSCs are angiogenin (ANG), angiopoietin-1 (ANGPT1), colony-stimulating factor (CSF), dipeptidyl peptidase IV (DPPIV), endothelin-1 (EDN1), interleukin- 8 (IL-8), insulin-like growth factor binding protein-3 (IGFBP3), monocyte chemoattractant protein-1 (MCP-1), and urokinase-type plasminogen activator (uPA) (Janebodin et al., 2013).

Additionally, stem cells from pulps of human exfoliated deciduous teeth (SHED) have bone marrow-derived MSC-like characteristics. An *in vivo* transplantation study showed that SHED could produce dental pulp-like tissue with the beneficial paracrine effects that are involved in immunomodulation and angiogenesis; characteristics that will enhance the clinical potential of dental mesenchymal stem cells to treat a variety of pathologies (Miura et al., 2003; Werle et al., 2016; Zhang et al., 2016). Although SHED are derived from deciduous teeth, the developmental and anatomical similarities between the deciduous and adult dental pulps has shown that SHED may also have perivascular origins and thus pericyte-like characteristics. Indeed, *in-vivo* studies have demonstrated that SHED were able to form functional vessel-like structures upon transplantation (Kim et al., 2016).

While DPSCs lose their plasticity through passaging, several investigations have provided enough evidence that SHED can retain their characteristics. Results from DNA microarray analysis revealed that there were 4386 genes that were expressed differently between DPSCs and SHED by 2.0-fold or more. SHED expressed higher levels of genes related to pluripotency (OCT4, SOX2, NANOG, and REX-1), cell proliferation, and extracellular matrix, including several cytokines such as fibroblast growth factor and tumor growth factor b (Kerkis and Caplan, 2012; Kaukua et al., 2015; Majumdar et al., 2016).

Studies in recent years have resulted in the recognition of a paracrine function of stem cells, and have suggested that stem cell transplantation may also be regarded as cell-based cytokine therapy. Exogenously delivered DPSC and SHED secrete factors, such as vascular endothelial growth factor, chemokine stromal cell-derived factor-1, nerve growth factor, brain-derived neurotrophic factor, and glial cell-derived neurotrophic factor which highlight their possible mechanism of function. The degree of cytoprotection offered by human DPSCs and conditioned media of human DPSCs was significantly greater compared with human MSCs and conditioned media of human MSCs, suggesting that human DPSCs could be a better therapeutic source for cerebral ischemia. The mechanism underlining this effect was that conditioned media of pulp CD31–side population cells significantly enhanced migration, anti-apoptosis and angiogenesis while having little effect on cell proliferation compared with the non-pulp-derived conditioned media. The proof-of-this concept was documented recently as an inductive microenvironment reconstituted from EDTA soluble chemical components of extracted teeth and the conditioned media of mobilized population DPSCs promoted cell proliferation, migration, and odontoblastic differentiation (Inoue et al., 2013; Hayashi et al., 2015; Song et al., 2015; Kawamura et al., 2016).

Recent work has additionally developed the methodology of intravenous administration of allogeneic dental pulp derived neurosphere cells as an important base for regenerative medicine and indicated that those cells were able to ameliorate the outcomes in focal brain ischemia in rat global cerebral ischemic condition. Since pulp stem cells can be obtained from exfoliated deciduous teeth as SHED or impacted adult wisdom teeth thus, preserved autologous or allogeneic dental pulp could be an attractive source for future regenerative therapy for oral diseases as well as systemic diseases (Kumasaka et al., 2017).

#### Dental mesenchymal stem cells: hope for a multitude of systemic ailments?

Being able to bank DPSCs obtained from adult third molars after routine extraction or bank SHED, has paved the road to many investigations as a powerful autologous stem cell source for treatment of a variety of ailments. Through gene and protein expression, DPSCs were shown to differentiate effectively into keratocytes *in vitro*, and to function *in vivo* without eliciting overt rejection. As a matter of fact, other investigators have provided evidence of DPSC-special characteristics that are similar to epithelial and neural stem cells even more than those of BMMSCs (Karaoz et al., 2011; Garzon et al., 2015). DPSCs have been shown to effectively differentiate into keratocytes confirmed by their expression of keratocyte-specific molecules similar to the normal human cornea. This evidence strongly points to the potential translation of DPSCs as an autologous cell source for targeting corneal regeneration (Syed-Picard et al., 2015).

Corneas are the most frequently transplanted tissue worldwide, however, this procedure is successful only if the recipient has a functional stem cell population. As a result, this limits what can be done for those patients with damaged limbal stem cell niches and who have developed limbal stem cell deficiencies because the corneal epithelial layer is no longer maintained (Ricardo et al., 2013). Recent studies have looked at restoring the stem cell population in a way to re-establish a functional limbus (Rahman et al., 2009). Contact lenses were used by many studies not only to correct vision or aesthetics, but also to provide an adequate reservoir for drugs and/or cells to adjust the environment of the cornea (Espandar et al., 2014; Bobba and Di Girolamo, 2016). In a revolutionary step toward routine clinical application, soft contact lenses were used as carriers to deliver DPSCs to enhance corneal epithelium regeneration. Investigators described that the cell/ scaffold construct once transplanted *in-vivo*, DPSCs had transferred from the contact lenses to the corneal surface and began to express the corneal specific markers cytokeratins 3 and 12. In addition to the expression of these markers, the DPSCs were able to prevent conjunctival cells from growing in the central cornea (Yam et al., 2015; Kushnerev et al., 2016).

Recently DPSCs have been shown to differentiate toward specific neuronal fates upon specific chemical and environmental cues. Co-culturing of DPSCs with rat retinal explants allowed DPSCs to enter into a retinal neuronal fate with upregulated brain-derived neurotrophic factor and retinal markers expression along with induction of the mature photoreceptor gene. From these studies it was suggested that DPSCs could be an enticing source of retinal-like stem cells that can differentiate into retinal neurons and even photoreceptors. This has paved the road to a novel strategy for reversing retinal degeneration (associated with age-related macular degeneration, retinitis pigmentosa, macular dystrophy, artery, or vein occlusion) (Bray et al., 2014; Syed-Picard et al., 2015; Yam et al., 2015).

Dental mesenchymal stem cells also hold great promise for the treatment of diabetes. Indeed, researchers have spent decades trying to find a reliable method to restore functional pancreatic islets that produce insulin. Embryonic or adult bone marrow stem cells have been shown to contribute to b-cell renewal and may help reverse nervous system damage (Ianus et al., 2003; Schonhoff et al., 2004; Lee et al., 2006; Koblas et al., 2009; Okura et al., 2009). It has been previously shown that type I diabetes could be cured by transplantation of insulin-producing islet cells from a donor pancreas. However, a critical limitation of such an approach is the lack of sufficient donor organs and the side-effects of immunosuppression which have hindered the application of such a therapy and have directed huge efforts to search for alternative sources of islet cells (Shapiro et al., 2000).

Major advances have been made in the understanding of developmental endocrinology that have opened the arena to stem cell therapy via differentiation of mesenchymal stem cells into pancreatic islets. Although there are many sources for MSCs such as umbilical cord blood, bone-marrow, and adipose stem cells that have the potential to differentiate into insulin-producing cells, these sources maybe scarce and still require invasive procedures to obtain them; factors which again have limited their use (Ianus et al., 2003; Lee et al., 2006; Koblas et al., 2009; Okura et al., 2009). Accordingly, DPSCs may be considered as an appealing source for MSCs, since their use is not controversial, they are easily accessible and available, and they pose no risk of discomfort for the patient. Researchers have transplanted DPSCs and demonstrated for the first time that these cells have a powerful and long lasting irreversible anti-nociceptive effect in mice with diabetic neuropathic pain. They showed that this effect was hardly reached by clinically used analgesics and could be explained by the cells' strong secretion of anti-inflammatory, angiogenic, and neurotrophic factors (Guimaraes et al., 2013; Izumoto-Akita et al., 2015).

Reports have demonstrated many common features among pancreatic β-cells of endodermal origin and those of neurons of ectodermal origin. During pancreatic development, the neural crest is linked with the pancreatic epithelium via Phox2b and Nkx2.2 which form a non-cell-autonomous feedback loop. This loop also regulates the proliferation of the beta-cell population, and thereby impacts insulin-secretory capacity and homeostasis of energy. Although, the neural crest cells do not differentiate into pancreatic islet cells, these cells do contribute indirectly to islet development via a novel non-cell autonomous negative-feedback interaction between the neural crest cells and the pancreatic cells (Schonhoff et al., 2004; Nekrep et al., 2008).

The previously established intrinsic similarities between neural cells and DPSCs, have suggested that stem cells of dental origin may offer a useful alternative to generate insulin producing cells. Studies have additionally documented the potential of dental mesenchymal stem cells derived from deciduous teeth or periodontal tissues to differentiate into insulin producing cells as a non-invasive source of cells for islet generation that can be used for autologous transplantation for the treatment of children with type I diabetes without risk of rejection and calls for banking deciduous teeth for clinical application (Govindasamy et al., 2011; Lee et al., 2014).

Recent work has also indicated that cryopreserved (Cryo-) DPSCs displayed comparable capacity for proliferation and differentiation as fresh-DPSCs. Cryo-DPSC transplantation was as effective as the transplantation of fresh-DPSCs in improving nerve conduction speed and blood flow in diabetic rats. DPSCs showed anti-inflammatory properties as they could ameliorate diabetic polyneuropathy upon transplantation into diabetic rats. These effects were apparent via their ability to modulate the proportions of M1/M2 macrophages in diabetic peripheral nerves (Hata et al., 2015; Omi et al., 2016). As it was mentioned before, SHED express many genes encoding extracellular and cell surface proteins at levels that are 2-folds higher than those in human bone marrow-derived MSCs (Sakai et al., 2012; Feng et al., 2013; Yamagata et al., 2013; Fafian-Labora et al., 2015; Yamamoto et al., 2016). Current data additionally support the fact that treatment with SHED-conditioned media (CM) can directly improve both survival and function of pancreatic β-cells much more than with Bone marrow-CM indicating anti-diabetic effects of the secreted paracrine factors of SHED-CM. These results have opened a new avenue for the treatment of diabetic patients (Izumoto-Akita et al., 2015).

Further important applications of dental mesenchymal stem cells are for treatment of kidney injuries. The anti-inflammatory ability of SHED secreted factors has been shown to promote the proliferation and migration of tubular epithelial cells through a paracrine mechanism in acute kidney injury (Hattori et al., 2015). Another very promising emerging application is in cardiac repair indicating that DPSC transplantation improves regional contractility as well as preventing ventricular remodeling. Ultra-structural analysis of Left Ventricular wall peri-infarct zones showed an increase in cardiomyocyte bundles that reduced infarcted area and a higher proportion of myofibroblasts. It is believed that DPSCs are able to repair infarcted myocardium, due to their ability to secrete pro-angiogenic and anti-apoptotic factors (Gandia et al., 2008). In a recent investigation, SHED-CM protected the heart from acute ischemic injury as it suppressed inflammation and apoptosis. SHED-CM antiapoptotic action was more effective than bone marrow-derived stem cell (BMSC)-CM or adipose-derived stem cell (ADSC)-CM in cardiac myocytes. SHED-CM could attenuate myocyte apoptosis representing an important therapeutic target for ischemic heart disease (Yamaguchi et al., 2015).

Recently, various stem cell populations have also been used to treat muscular dystrophy. The ability of these cells to mobilize host progenitor cells and promote angiogenesis is partly owed to their ability to resist stress but mainly due to their paracrine-mediated effects which directly caused improvement of muscle histopathologies (Gharaibeh et al., 2011). In this aspect, human DPSCs have also been employed (Martinez-Sarra et al., 2017) and they have shown capability of supporting the improvement of skeletal muscle regeneration by promoting new blood vessel formation and interacting directly and indirectly with myoblasts as well as promoting the proliferation of myogenic precursor cells within the transplanted muscles. The engraftment of these cells contributed reducing fibrosis within the dystrophic muscle, which further enhanced regeneration (Pisciotta et al., 2015).

Another promising application for dental mesenchymal stem cells was the ability to differentiate DPSCs into bladder-associated smooth muscle cells (SMCs) when treated with a combination of conditioned media from bladder SMCs and TGF-β1 (Song et al., 2016). Furthermore, although DPSCs rarely express tendon-related proteins *in vitro*, such as SCX, EYA2, and TNC; when cultured in 2-D culture dishes researchers have been able to demonstrate that DPSCs could be differentiated into tenogenic-like cells and form more mature tendon-like tissues under mechanical loading in the mouse model (Chen Y. Y. et al., 2016).

## The road to translational/ precision regenerative medicine

The field of *precision medicine* which includes “prevention and treatment strategies that take individual variability into account” has been dramatically improved by the recent development of large-scale biologic databases and the advent of computational tools for analyzing large quantities of data. At the forefront of this new era of medicine is the discovery of the human genome sequence that has generated knowledge applicable to span all of health and disease (Collins, 2015; Collins and Varmus, 2015).

The discovery of stem cells has not only revolutionized the study of disease, but also holds major therapeutic value. Through combining stem cell research with new methods to efficiently generate targeted mutations in mammalian cells, a new window for precise diagnosis and treatment has now been opened. The twenty-first century can truly be defined as the century of biology with investments in biomedical research which will optimally have its rewards on both economy and health. The main objective of biomedical research is to speed up the clinical translation of basic research and eventually to therapeutic strategies to reduce human suffering and minimize health inequalities (Cong et al., 2013; Surkis et al., 2016).

The evolution of patient-specific medicine has witnessed strides of achievement with the dawn of technologies that can reprogram human adult somatic cells into pluripotency or into a different cell type making the study of patient-specific cells a possibility. Having patient-specific cells allows researchers to thoroughly understand the disease mechanisms and also provide a platform for drug testing which could then be followed by upscaling these strategies. There are a handful of stem-cell-based treatments available, such as bone marrow transplantations to treat blood disorders, which have proven beneficial for patients in well-designed clinical trials and are now offered as treatments. In clinical trials, most therapies that utilized adult stem cells and have shown successful results, are shifting to using DPSCs for the same application and have started to show promising results. Clinicians and researchers have demonstrated that 75% of patients with limbal stem-cell deficiency who received limbal stem-cell grafts as corneal transplants, showed successful treatment with follow-up extending up to 10 years (Rama et al., 2010; de Araujo and Gomes, 2015). However, because of the concern of removing the limbus from a healthy eye, with the potential harm to limbal epithelial stem cells during isolation in addition to disruption occurring to the native limbal niche, different protocols have arisen to modify the original technique (Tseng et al., 2010; Ramirez et al., 2015).

With a continuously growing body of evidence demonstrating that the trophic effects of proteins and paracrine factors rather than terminal engraftment are key factors, regenerative medicine appears to offer a factual approach toward transforming healthcare in a true clinical sense. Yet, there are currently many challenges facing the use of cellular products. In most clinical situations, the number of primary donor cells needed for transplantation maybe insufficient to meet the clinical therapeutic level needed and the expansion of cells in culture under good manufacturing process (GMP) conditions is required to address this shortage (Heathman et al., 2015). Autologous cell-based therapies are based on personalized (service-based) medicine in addition to the complexity of upscaling production and delivery of a cost effective autologous cell-based therapy. In fact, delivery of autologous cell-based products can be complex due to the inability to perform long term preservation for transport and delivery (Mason and Dunnill, 2009). Autologous therapies requiring participation by clinicians; partly because of the need for initial patient biopsy and a subsequent surgical restoration phase; have resulted in a smaller scale of clinical trial studies since clinician participation is restricted to autologous procedures. High levels of process and product characterization are required for autologous cell therapies making the establishment of allogeneic cell banks a much more cost effective option (Dodson and Levine, 2015).

Transplantation of hematopoietic stem cells has illustrated success for over 50 years since 1960 and is becoming the standard-of-care for numerous indications. Currently, tissue-specific stem cells have focused on the paradigm shift of replacement of diseased tissue with autologous or allogeneic stem cells (Korbling and Estrov, 2003; Boo et al., 2011).

However, research has shown that the engraftment of implanted cells may not be all that crucial thus opening a new avenue of clinical application for mesenchymal stem cells in immune- and inflammation-mediated diseases. This therapeutic approach relies on indirect effects of mesenchymal stem cells in addition to their direct effects (Griffin et al., 2013; Wang et al., 2016). In turn, this novel direction has urged the shift from autologous to allogeneic cell sources. While the immunomodulatory properties of MSCs were identified few years ago, nearly half “almost 42%” of all registered clinical trials are being conducted for immune-/inflammation mediated diseases with only 32.5% that use autologous sources and over 50.9% of trials using allogeneic sources. It is becoming obvious that the capacity to use allogeneic MSCs has greatly contributed to increased popularity of stem cells (Griffin et al., 2013; Introna et al., 2014; Wang et al., 2016; NIH^1^).

The current approach of using autologous stem cells is to avoid immune rejection of donor cells that is probable after allogeneic transplantation. However, in spite of promising results, harvesting autologous cells is not without its drawbacks. These include logistic, economic, and timing constraints particularly since the target population for these therapies is likely the elderly who already present for treatment with multiple complex disorders. As previously mentioned there are a number of studies that have shown that mesenchymal stem cells (MSCs) obtained from elderly donors, and those with diabetes may have limited therapeutic potentials. This has opened the avenue for the obvious clinical advantages of universal donor cells from young healthy individuals which could be used for stem cell allotransplantation without the need for immunosuppression. Additionally, serious consequences of replicative senescence in hMSCs are that this phenomenon may impair the secretion of soluble factors in response to the inflammatory microenvironment thus resulting in loss of the immunoregulatory functions of these cells (Zhuo et al., 2010; Bustos et al., 2014).

A series of observations has highlighted the immunomodulatory properties of allogeneic MSCs whereby they are able to survive and differentiate when transplanted in immunecompatible-mismatched recipients. They do not induce a proliferative T-cells response but instead can modulate their function in allogeneic experiments indicating that the hypoimmunogenic pattern is a unique expression pattern of cell-surface antigens on MSCs (Horwitz et al., 2002; Tse et al., 2003; Atoui and Chiu, 2012). Hence, allogeneic “off-the-shelf” therapies maintain long-term stability and by automating the manufacturing process enhanced processing and control can be achieved that dictate the introduction of the universal cell donor and paint the next generation of clinical cell-based therapy trials (Kinkaid et al., 2010; Telukuntla et al., 2013; Heathman et al., 2015).

## Translational regenerative dentistry for the future

The presence of active immune responses in the oral cavity make it a challenging unique environment which inevitably affects periodontal tissue, jaw bone, and tooth regeneration. Due to the highly dynamic inflammatory environment of the oral cavity; dental MSCs, including SHED, SCAP, periodontal ligament stem cells (PDLSCs), and jaw bone MSCs, have shown strong immunomodulatory capacities (Wada et al., 2009; Yamaza et al., 2010, 2011; Ding et al., 2010a; Hieke et al., 2016; Rajan et al., 2016). For example, DPSCs can suppress T lymphocyte growth rate by 18% higher than BMMSCs, indicating possible superior immunosuppression properties of DSCs (Taşl et al., 2016).

More importantly, DPSCs can strongly inhibit proliferation of peripheral blood mononuclear cells induced by mitogens in comparable degrees to PDLSCs yet stronger than BMSCs. This effect appears to be modulated by secretion of transforming growth factor-b1. Earlier reports have proven that the addition of DPSCs can inhibit the T-cell response up to 91% while BMSCs allowed only 75% inhibition of T-cell response, highlighting the possible use of DPSCs in different individuals or to be used for immune therapy (Pierdomenico et al., 2005; Ding et al., 2015). The dental pulp of healthy young patients appears to possess the generic MSC phenotype and can strongly inhibit acute allogeneic immune responses resulting from T-lymphocyte stimulation again through their release of TGF-β (Kwack et al., 2017). This provides insight into the potential clinical use of hDPSCs for dental and non-dental tissue regeneration using allogeneic transplantation, that will move the field of stem cell therapy into true clinical application faster than before.

Human dental pulp stem cells (DPSC) are an attractive option for exogenous stem cell therapy and have several advantages for translational cell-based therapy. Teeth are a clinically accessible source especially since many older adults possess their own teeth which could then be used as a source of viable human DPSCs for autologous transplantation. The feasibility that viable DPSCs can still be obtained up to 120 h of tooth storage in phosphate buffered saline (PBS) at 4°C post extraction from a whole tooth and can be obtained from teeth undergoing root canal treatment without the need for extraction; all support the possibility for banking these tissues for regenerative medicine applications (Arthur et al., 2008; Perry et al., 2008; Huang A. H. et al., 2009). This is in addition to the low immunogenic response of DPSCs which could open the door for allogeneic cell grafts. Moreover, as previously mentioned human DPSCs have been shown to have neurogenic potential and to generate functional neurons in addition to their high proliferative capacities (Gronthos et al., 2000; Leong et al., 2012).

Clinical banking of DPSCs can be facilitated by storing teeth that have been subjected to minimal processing which also minimizes cost particularly if these cells are not planned for immediate expansion and use (Papaccio et al., 2006; Woods et al., 2009). Additionally, expanded cells can be stored for a minimum of 6 months and even longer at −85°C. SHED isolated from deciduous pulp tissues cryopreserved for over 2 years can maintain their stem cell properties in a way comparable to SHED isolated from fresh tissues (Papaccio et al., 2006; Woods et al., 2009; Ding et al., 2010b; Ma et al., 2012). Indeed, there is an international call for banking SHED as they require less than one third of the cost of cord blood storage and they are not subject to the same ethical concerns as embryonic stem cells. SHED may also be useful to a certain extent to their immediate family and blood relatives such as grandparents, parents, uncles, and siblings. SHED cells are complementary to stem cells from cord blood (Arora et al., 2009). Although, bone marrow and adipose tissue are considered potential sources of stem/progenitor cells; painful collection protocols, the decline of the amount of stem/progenitor cells with age, the necessity for general anesthesia, the reduced proliferation capacity, and risk of morbidity at the collection site have all encouraged the search for alternatives such as dental mesenchymal stem cells (Huang G. T. et al., 2009; Davies et al., 2015).

Several protocols have been developed to obtain clinical-grade dental pulp stem/progenitor cells (DPSCs) from teeth while avoiding any alteration of their biological properties and preserving the quality of the derived cell-based products. Cell performance is affected by cell isolation and expansion conditions and thus optimization and standardization procedures for MSC-based product manufacturing are required (Menard et al., 2013; Menard and Tarte, 2013; Huang and Garcia-Godoy, 2014; Pacini, 2014). While the cell-based approach using dental mesenchymal stem cells has already laid down the proof-of-the-concept in regenerative medicine since the discovery of DPSCs in the year 2000; developing standardized cGMP protocols requires tremendous effort regarding several key players as its safety via clinical trials, increased efficiency and reproducibility, and precise cost estimation after all these regulatory processes (Eubanks et al., 2014; La Noce et al., 2014b; Ducret et al., 2015a).

In a more recent study, specific conditions for hDPC isolation, storage, and amplification with a medicinal manufacturing approach have been developed. These conditions include minimal tissue manipulation, enzyme-free isolation, use of xenogeneic-free products, and serum free media culture. They allow for the expression of stem/progenitor cell markers and preserve amplification kinetics without inducing karyotype abnormality while maintaining the differentiation potential of DPSCs into osteoblast/odontoblast cells. Although there are many successful advanced therapies with medicinal products and good regulations from the FDA “21 CFR Part 1271” and the European Medicines Agency (European Directive 1394/2007), further studies are required to determine more specific properties that could be used for the regeneration of human tissues, with standardized cell-based medicinal products (Ducret et al., 2015b, 2016).

**Figure 1:**
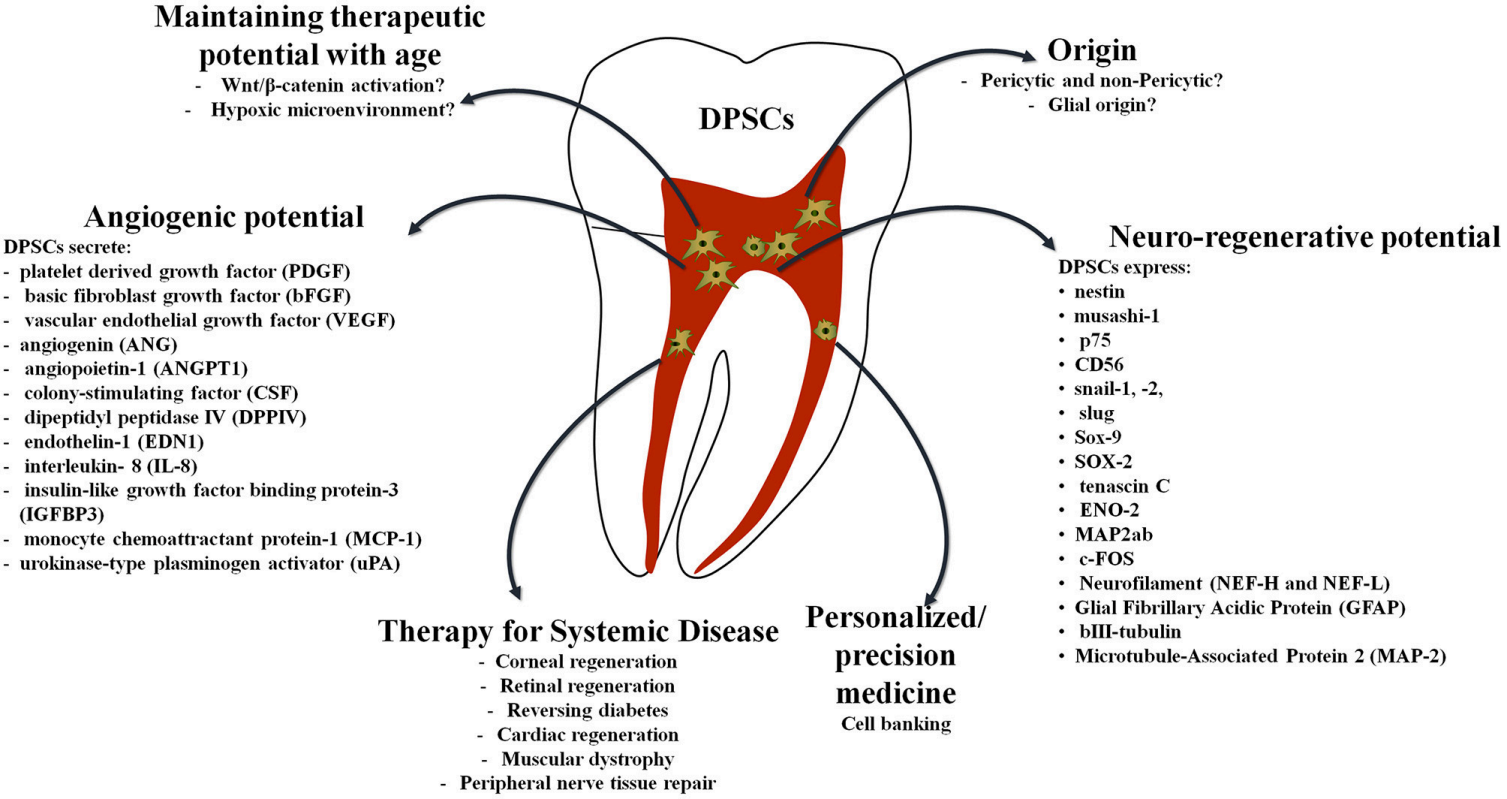
Translational regenerative potential of dental pulp stem cells.

In conclusion, as the fields of regenerative medicine and dentistry quickly move into an era of personalized and precision therapeutics, the use of dental pulp stem cells for clinical translation will soon become a feasible reality (Figure 1). Dental pulp stem cells represent a versatile and readily available source of stem cells that can be stored efficiently for long periods of time. Dental pulp stem cells not only offer potential treatment for many dental dilemmas but their use may span numerous non-dental applications as well (Table 1). This is an addition to their therapeutic benefits that are elicited not only via their exogenous transplantation but through their paracrine-mediated mechanisms as well. With the development of standardized and optimized clinical good manufacturing process guidelines, both allogenic and autologous use of these cells for therapy may soon become an achievable goal.

**Table 1:** A summary of the dental and non-dental therapeutic applications of dental pulp stem cells.

**Dental therapeutic applications**	**Non-dental therapeutic applications**	**Benefits**	**Limitations**
Dentin/Pulp complex regeneration	Spinal cord injury and peripheral nerve tissue repair	Clinically accessible source	May become limited with age
Craniofacial bone regeneration	Cardiac repair/angiogenesis and treatment of ischemic disease	Can be banked easily and with low cost	Lack of optimized standardized protocols for isolation and characterization of clinical grade cells
Enhancing osseointegration of dental implants	Corneal and retinal regeneration	Higher proliferative and osteogenic differentiation capacities compared to bone marrow mesenchymal stem cells	DPSCs may lose plasticity with passaging as compared to SHED which retain their characteristics
Treatment of osteoradionecrosis	Muscular dystrophy and tendon regeneration	Derived from the neural crest	Express pluripotency markers at a lesser degree than SHED
	Diabetes mellitus including treatment of diabetic neuropathic pain		

## Author contributions

All authors listed have made a substantial, direct and intellectual contribution to the work, and approved it for publication.

### Conflict of interest statement

The authors declare that the research was conducted in the absence of any commercial or financial relationships that could be construed as a potential conflict of interest.

## Footnotes

^1^NIH Clinical Trial Database. Available online at: https://clinicaltrials.gov/ct2/results?term=allogeneic+mesenchymal+stem+cells&Search=Search
